# Albert de la Chapelle (1933–2020)

**DOI:** 10.1038/s41431-021-00863-4

**Published:** 2021-03-24

**Authors:** Helena Kääriäinen, Kristiina Aittomäki

**Affiliations:** 1 Finnish Institute for Health and Welfare, Helsinki, Finland; 2 University of Helsinki, Helsinki, Finland

**Keywords:** Endocrinology, Cancer


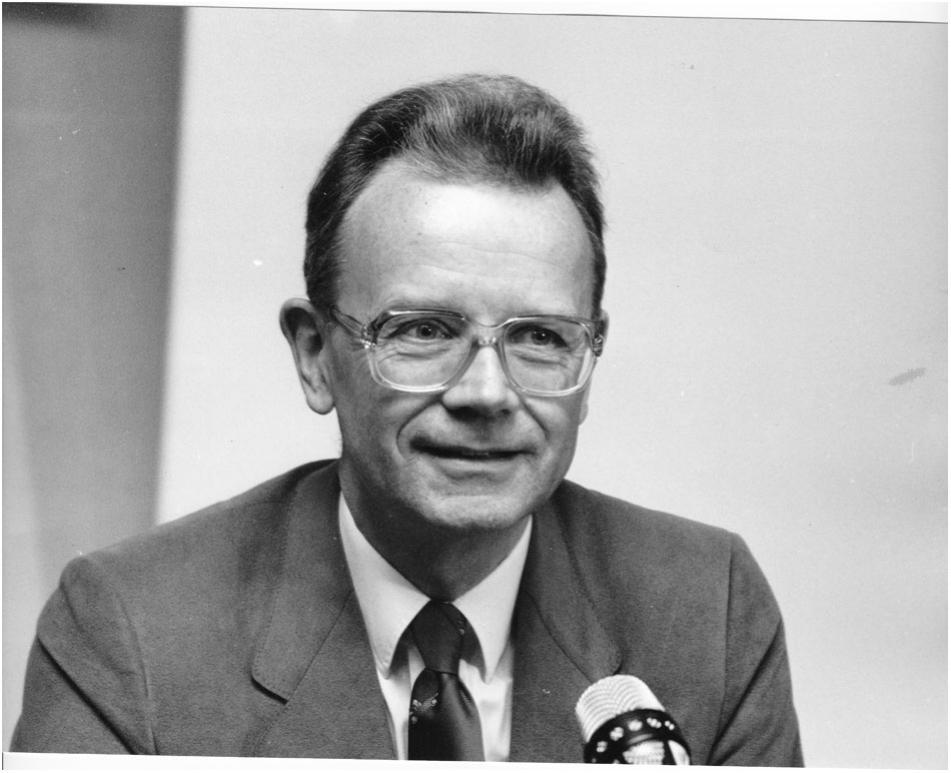
Professor Albert de la Chapelle, Emeritus Professor of Human Genetics at the University of Helsinki and Distinguished University Professor and Cancer Scholar at The Ohio State University passed away on 10th December 2020, after a short illness.

Albert was born 1933 in Helsinki, Finland, and spent his childhood at the country estate of his family in South-Western Finland. His early school years were complicated by the war (in Finland 1939–1944) but he still graduated from high school (“gymnasium”) one year earlier than usual. He studied medicine at the University of Helsinki and started specialty training for internal medicine at Helsinki University Hospital. This led him to become interested in endocrinology. That was the time when first discoveries in chromosomal studies were published. Albert started a chromosome laboratory studying at first sex chromosome disorders and mechanisms of sex determination. Later, when chromosome banding techniques came to use, his interest moved toward chromosomal changes in hematologic malignancies. When the monogenic diseases belonging to the Finnish Disease Heritage were delineated one after the other, Albert and his group concentrated in these diseases clarifying the molecular basis of over a dozen of these disorders. Mapping and cloning the diastrophic dysplasia gene and characterizing the Finnish founder mutation by Albert’s group was an example of one of the first positionally cloned genes. In recent years Albert is maybe the best known for his role in determining the molecular basis of hereditary cancer, notably the role of the mismatch repair genes in Lynch syndrome. Together with his group he detected the phenomenon of microsatellite instability in hereditary cancer.

Since the beginning of his career, he formed active collaborations in Finland and abroad. Whenever new techniques were developed, he would travel or, later, send one of his students to learn the methods in the most advanced laboratories. He had a deep respect for skillful clinicians and involved them closely in his genetics studies, to guarantee exact phenotyping and also clinical understanding for translation of these molecular events into clinical work.

Albert’s major honors include: Memberships in the Academy of Finland, the Royal Swedish Academy of Sciences, and the US National Academy of Sciences.

Albert was an important figure in forming collaboration between European geneticists. European Society of Human Genetics (ESHG) was established on March 15, 1967, and Albert was one of the outstanding European Geneticists involved in this initiative. He was also actively involved some 20 years later in the reform of the Society which began to develop 1988 at the ESHG meeting in Cardiff and was completed in 1991 at the Leuven meeting when ESHG got its first elected president. Albert served in ESHG as a Board member 1966–1995, Chairman of the Aims and Statutes Committee 1990–1991, and President 1993–1994. He also was Associate Editor of *European Journal of Human Genetics* for several years and was presented with the Mauro Baschirotto Award in 2002.

Albert had a deep lifelong interest in visual art. His remarkable art collection consists of traditional, mainly Finnish art from nineteenth and twentieth centuries as well as highly interesting contemporary paintings and sculptures. Already some years ago, together with the city Tammisaari near to his childhood home, the Albert de la Chapelle Art Foundation was created. This enables the city to build an art museum where his art collections, donated to the Foundation, but also other exhibitions will be presented.

Professor Albert de la Chapelle was the originator of medical genetics in Finland and his many students and colleagues remember him as an inspiring and encouraging teacher and a memorable friend. His passion for science was reflected in his habit known by all his Ph.D. students. This was to light a candle, whenever wonderful things were discussed, and this was always when you discussed your own project with him. At the time of dissertation, he presented his students with a silver candle holder to keep the flame burning.

